# The phosphatidylinositol 3-kinase/Akt/mTOR signaling network as a therapeutic target in acute myelogenous leukemia patients

**DOI:** 10.18632/oncotarget.114

**Published:** 2010-05-27

**Authors:** Alberto M. Martelli, Camilla Evangelisti, Francesca Chiarini, James A. McCubrey

**Affiliations:** ^1^Department of Human Anatomical Sciences University of Bologna, Bologna, Italy; ^2^IGM-CNR, Sezione di Bologna c/o I.O.R., Bologna, Italy; ^3^Department of Microbiology & Immunology, School of Medicine, East Carolina University, Greenville, NC 27834, USA

**Keywords:** PI3K/Akt/mTOR, leukemia, leukemic stem cells, signal transduction modulators, targeted therapy, combination therapy

## Abstract

The phosphatidylinositol 3-kinase (PI3K)/Akt/mammalian target of rapamycin (mTOR) signaling axis plays a central role in cell proliferation, growth, and survival under physiological conditions. However, aberrant PI3K/Akt/mTOR signaling has been implicated in many human cancers, including acute myelogenous leukemia (AML). Therefore, the PI3K/Akt/mTOR network is considered as a validated target for innovative cancer therapy. The limit of acceptable toxicity for standard polychemotherapy has been reached in AML. Novel therapeutic strategies are therefore needed. This review highlights how the PI3K/Akt/mTOR signaling axis is constitutively active in AML patients, where it affects survival, proliferation, and drug-resistance of leukemic cells including leukemic stem cells. Effective targeting of this pathway with small molecule kinase inhibitors, employed alone or in combination with other drugs, could result in the suppression of leukemic cell growth. Furthermore, targeting the PI3K/Akt/mTOR signaling network with small pharmacological inhibitors, employed either alone or in combinations with other drugs, may result in less toxic and more efficacious treatment of AML patients. Efforts to exploit pharmacological inhibitors of the PI3K/Akt/mTOR cascade which show efficacy and safety in the clinical setting are now underway.

## INTRODUCTION

Acute myelogenous leukemia (AML) is a highly heterogeneous group of malignant clonal diseases characterized by deregulated proliferation of hematopoietic stem cells and myeloid progenitors. This results in accumulation, in the bone marrow, of myeloid cells with an impaired differentiation program and resistant to cell death. AML accounts for about 80% of adult leukemias and is a disorder of the elderly, with a median age at diagnosis of 65 years and a growing incidence over 65 years [1]. Most AML cases respond well to initial polychemotherapy, but disease relapse occurs in the large majority of patients. The standard therapeutic approach for AML patients is high-dose polychemotherapy, consisting of cytarabine and an anthracycline antibiotic like daunorubicin or idarubicin, or the anthracendione mitoxantrone [2]. While results of AML treatment have improved in younger patients who can tolerate intensified treatment strategies, there have been limited changes in outcome among individuals who are older than 60 years. Therefore, the prognosis of AML remains severe, with an overall 5-year survival rate around 20%, despite continuous advances in our understanding of AML biology. Furthermore, patients with AML arising out of myelodysplastic syndrome or who are older than 60 years have an even worse prognosis (<10% survival at 5 years) [3]. Therefore, there remains a need for innovative, rationally designed, minimally toxic, therapies for AML, especially for the elderly [4].

Only one subtype of AML, acute promyelocytic leukemia (APL), displays a much better prognosis, as differentiation therapy with arsenic trioxide or all-*trans* retinoic acid (ATRA), used alone or in combination with chemotherapeutic drugs, has proven quite successful in APL patients [5]. It is now clear that a hierarchical organization of the hematopoietic system does exist in AML, as in normal hematopoiesis. Indeed, AML is initiated and maintained by a small, self-renewing population of leukemic stem cells (LSCs), which give rise to a progeny of more mature and highly cycling progenitors (colony forming unit-leukemia, CFU-L). CFU-Ls do not self-renew, however they are committed to proliferation and limited differentiation. By doing so, they originate a population of blast cells which constitute the majority of leukemic cells in both the bone marrow and peripheral blood of patients. The exact phenotype of LSCs is still debated, but they are comprised in the CD34+/CD38^−/low^ population [6]. The majority of LSCs are quiescent and insensitive to traditional chemotherapeutic drugs. This latter feature explains, at least in part, the difficulties in eradicating this cell population by conventional polychemotherapy. Thus, novel therapeutic strategies for AML eradication should also target LSCs [7]. In AML, aberrant activation of several signal transduction pathways strongly enhances the proliferation and survival of both LSCs and CFU-Ls [8, 9]. Therefore, these signaling networks are attractive targets for the development of innovative therapeutic strategies in AML [10].

The phosphatidylinositol 3-kinase (PI3K, a family of lipid kinases)/Akt/mammalian target of rapamycin (mTOR) signaling cascade is crucial to many widely divergent physiological processes which include cell cycle progression, transcription, translation, differentiation, apoptosis, motility, and metabolism [11]. However, the PI3K/Akt/mTOR signaling pathway represents one of the major survival pathways that is deregulated in many human cancers and contributes to both cancer pathogenesis and therapy resistance. Over the last few years, it has been reported that constitutive activation of the PI3K/Akt/mTOR signaling network is a common feature of AML patients [12]. Furthermore, pathway activation confers leukemogenic potential to mouse hematopoietic cells [13]. Therefore, this signal transduction cascade may represent a valuable target for innovative therapeutic treatment of AML patients. The aim of this review is to give the reader an updated overview of the relevance of PI3K/Akt/mTOR signaling activation in AML patients and to focus on small molecules which will possibly have an impact on the therapeutic arsenal we have against this disease.

### The PI3K/Akt/mTOR pathway

#### PI3K

**Fig. 1.**
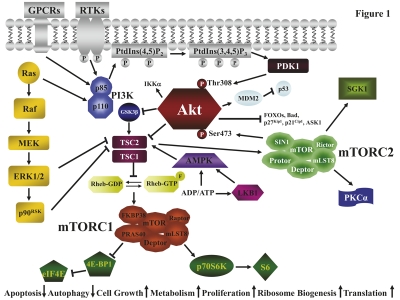
The PI3K/Akt/mTOR signaling pathway. GPCRs, RTKs, and Ras activate PI3K. PI3K generates PtdIns (3,4,5)P_3_ from PtdIns (4,5)P_2_. PtdIns (3,4,5)P_3_ attracts to the plasma membrane PDK1 which phosphorylates Akt on Thr308. Full Akt activation requires Ser473 phosphorylation which is effected by mTORC2. Most of the Akt substrates are inactivated by phosphorylation. Active Akt inhibits TSC2 activity through direct phosphorylation. TSC2 is a GAP that functions in association with TSC1 to inactivate the small G protein Rheb. Akt-driven TSC1/TSC2 complex inactivation allows Rheb to accumulate in a GTP-bound state. Rheb-GTP then activates the protein kinase activity of mTORC1. mTORC1 targets p70S6K and 4E-BP1 which are critical for translation. 4E-BP1 phosphorylation by mTORC1 results in the release of eIF4E, while p70S6K phosphorylates ribosomal S6 protein. The TSC1/2 complex is required to activate also mTORC2. However, other signaling cascades impinge on mTORC1, including GSK3β, the Ras/Raf/MEK/ERK1/2/p90^RSK^ pathway, and the LKB1/AMPK network which is sensitive to the ADP/ATP ratio. Arrows indicate activating events, whereas perpendicular lines indicate inhibitory events.

The family of PI3K enzymes is characterized by the ability to phosphorylate the 3′-OH group in inositol lipids and comprises three different classes, I, II, and III. Class I PI3K preferred substrate is phosphatidylinositol 4,5 bisphosphate [PtdIns (4,5)P_2_] which is phosphorylated to phosphatidylinositol 3,4,5 trisphosphate [PtdIns (3,4,5)P_3_] [14, 15]. PtdIns (3,4,5)P_3_ recruits to the plasma membrane pleckstrin homology (PH) domain-containing proteins, which include phosphoinositide-dependent protein kinase 1 (PDK1) and Akt. Class I PI3K is divided further into A [activated by receptor tyrosine kinases (RTKs), Ras, and G-protein coupled receptors (GPCRs)] and B (activated by GPCRs) subtype **(Figure 1)**. Class IA PI3Ks are heterodimeric enzymes composed of a regulatory (p85α, p85β, p55α, p55γ, p50α) and of catalytic (p110α, p110β, p110δ) subunits. Class IB PI3K comprises a p101 regulatory and a p110γ catalytic subunit [16]. Both p110α and p110β PI3K play fundamental roles during development, so that their homozygous knockout is embryonic-lethal [17]. In contrast, p110γ and p110δ PI3Ks are mostly related to the immune system functions, so that their knock-down leads to defective immune responses [18]. Class II PI3Ks, which comprise the PI3K-C2α, -C2β, and -C2γ isoforms, preferentially phosphorylate phosphatidylinositol to yield phosphatidylinositol 3 phosphate. Although class II PI3Ks are widely expressed in mammalian organs and tissues, their relevance in cell signaling and cancer biology is not clear at the moment [19].

Vacuolar protein sorting 34 (vps34) is the only class III PI3K and exists as a heterodimer bound to the vps15 regulatory subunit (previously referred to as p150 in mammals). Vps34 has been implicated in nutrient signaling, endocytosis, and autophagy [20].

Activating mutations in the gene coding for p110α (PIK3CA) have been found in many human cancer types, including tumors of the colon, brain, ovary, breast, liver, and stomach, and could at least partially explain pathway up-regulation in these neoplasms [21]. Nevertheless, in tumor models (brain, prostate, breast) driven by PTEN (phosphatase and tensin homolog deleted on chromosome 10) deficiency, knock-out of p110β, but not p110α, was required to inhibit Akt activation [17]. Wild-type p110α is not oncogenetic when overexpressed, whereas wild-type p110β, p110γ, and p110δ PI3Ks are oncogenetic when ectopically expressed in chicken fibroblasts [22]. Nevertheless, their contribution to oncogenesis is only beginning to emerge [23].

#### Akt

Akt, a 57-kDa serine/threonine protein kinase, is the cellular homolog of the *v-akt* oncogene. The Akt family comprises three highly conserved isoforms: Akt1/α, Akt2/β, and Akt3/γ, which display a high degree of sequence homology [14]. However, functional differences exist between Akt isoforms, as Akt2 is involved in insulin-mediated glucose uptake [24] and in cell motility/invasion/metastatic potential of cancer cells [25].

Akt contains an NH_2_-terminal PH domain, that interacts with PtdIns (3,4,5)P_3_. Once Akt is recruited at the plasma membrane, its activation loop is phosphorylated on Thr308 by PDK1 while the mTOR complex 2 (mTORC2) phosphorylates Ser473 in the Akt COOH-terminus **(Figure 1)**. Full Akt activation requires both the phosphorylation steps. Active Akt migrates to both the cytosol and the nucleus. Nuclear Akt may fulfil important anti-apoptotic roles [26]. Nevertheless, the relative contribution of Akt signaling at the plasma membrane, the cytosol, and the nucleus remains to be elucidated. However, it is intriguing that the protein promyelocytic leukemia (PML) is involved in the dephosphorylation of nuclear Akt as PML specifically recruits the Akt phosphatase, protein phosphatase 2A (PP2A), as well as phosphorylated Akt into PML nuclear bodies [27]. These bodies, however, are disrupted by the fusion protein, PML-RARα, which is the hallmark of APL [5, 28]. This could be one of the reasons for Akt activation which is detected in APL [29]. Thus, this finding highlights the growing importance of Akt compartmentalization in human cancer pathogenesis and treatment.

So far, over 100 Akt substrates have been identified [30]. Of these, about 40 which mediate the pleiotropic Akt functions have been characterized, including Bad, caspase-9, murine double minute 2 (MDM2), IκB kinase (IKK) α, proline-rich Akt substrate 40-kDa (PRAS40) 40, the FOXO family of Forkhead transcription factors, apoptosis signal-regulated kinase 1 [ASK1, a negative regulator of pro-apoptotic c-Jun N-terminal kinase (JNK)], Raf, p27^Kip1^, p21^Cip1^, glycogen synthase kinase 3β (GSK3κ. Each of these substrates has a key role in the regulation of cell survival and proliferation, either directly or through an intermediary [16, 31]. A rare, oncogenetic, activating mutation (E17K) in the PH domain of Akt1 has been detected in some types of solid cancers (breast, colon, ovary). This mutation resulted in Akt constitutive binding to the plasma membrane and was leukemogenic in mice [32].

#### mTOR

**Fig. 2.**
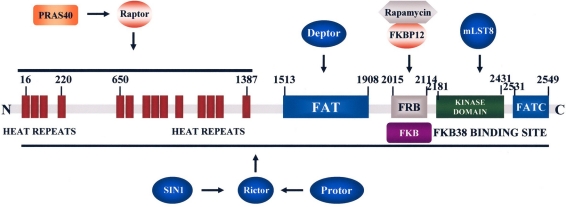
A schematic presentation of mTOR structure. Some of the proteins interacting with mTOR domains are highlighted. The FRB domain is where the FKBP12 and rapamycin complex binds which is within the region that binds FKBP38.

mTOR is an atypical 289-kDa serine/threonine kinase, originally identified in the yeast *Saccharomyces Cerevisiae*, that belongs to the PI3K-related kinase family and displays a COOH-terminal catalytic domain with a high sequence homology to PI3K **(Figure 2)**. This similarity could explain the cross-inhibition of mTOR by drugs which target PI3K (see below) [33]. mTOR signaling is conserved in eukaryotes from plants and yeasts to mammals. mTOR exists as two complexes, referred to as mTOR complex 1 (mTORC1) and mTORC2. mTORC1 is comprised of mTOR/Raptor/mLST8/PRAS40/FKBP38/Deptor and is sensitive to rapamycin and its derivatives (rapalogs). mTORC2 is composed of mTOR/Rictor/mLST8/SIN1/Protor/Deptor and is generally described as being insensitive to rapamycin/rapalogs, although long-term treatment of about 20% of cancer cell lines with rapamycin/rapalogs leads to dissociation of mTORC2 [34, 35]. mTORC1 signaling integrates environmental clues (growth factors, hormones, nutrients, stressors) and information from the cell metabolic status. Thus, mTORC1 controls anabolic processes for promoting protein synthesis and cell growth [36]. mTORC1 regulates translation in response to nutrients/growth factors by phosphorylating components of the protein synthesis machinery, including p70S6 kinase (p70S6K) and eukaryotic initiation factor 4E-binding protein 1 (4E-BP1). p70S6K phosphorylates the 40S ribosomal protein, S6, leading to active translation of mRNAs, while 4E-BP1 phosphorylation by mTORC1 on several amino acidic residues (Ser37; Thr46; Ser65; Thr70) results in the release of the eukaryotic initiation factor 4E (eIF4E). eIF4E is a key component for translation of 5′ capped mRNAs, which include transcripts encoding growth promoting molecules, such as c-Myc, cyclin D1, cyclin-dependent kinase 2, retinoblastoma protein, p27Kip1, vascular endothelial growth factor (VEGF), and signal activator and transducer of transcription 3 (STAT3) [34, 37]. Furthermore, mTORC1 negatively regulates autophagy, a non-apoptotic form of cell death, which is attracting much attention, as it could affect sensitivity of tumors (including leukemias) to various forms of therapy [38].

Akt-mediated regulation of mTORC1 activity involves several mechanisms. Akt inhibits TSC2 (Tuberous Sclerosis 2 or hamartin) function through direct phosphorylation. TSC2 is a GTPase-activating protein (GAP) which associates with TSC1 (Tuberous Sclerosis 1 or tuberin) for inactivating the small G protein Rheb (Ras homolog enriched in brain). TSC2 phosphorylation by Akt represses GAP activity of the TSC1/TSC2 complex, allowing Rheb to accumulate in a GTP-bound state. The mechanism by which Rheb-GTP activates mTORC1 has not been fully elucidated yet, although Rheb requires to be farnesylated for activating mTORC1 [39]. Thus, it could be inhibited by farnesyl-trasferase inhibitors (FTIs). Akt also phosphorylates PRAS40, an inhibitor of the interactions between mTORC1 and its substrates, and by doing so, prevents PRAS40 ability to suppress mTORC1 signaling [40]. Moreover, PRAS40 is a substrate of mTORC1 itself, and it has been demonstrated that mTORC1-mediated phosphorylation of PRAS40 facilitates the removal of its inhibition on mTORC1 [41].

Moreover, Ras/Raf/mitogen-activated protein kinase kinase (MEK)/extracellular signal-regulated kinase (ERK) 1/2 signaling positively regulates mTORC1 activity, as both ERK 1/2 and p90 ribosomal S6 kinase (p90^RSK^) phosphorylate TSC2, thus suppressing its inhibitory function on Rheb [42] **(Figure 1)**. mTORC1 signal transduction is inhibited by the master metabolic regulator, energy-sensing AMP-dependent protein kinase (AMPK), given that AMPK phosphorylates and activates TSC2 [43].

The mechanisms for mTORC2 regulation have only begun to be revealed. However, mTORC2 activation requires PI3K and the TSC1/TSC2 complex, but is independent of Rheb and is largely insensitive to either nutrients or energy conditions [44]. mTORC2 phosphorylates Akt on Ser473 which enhances subsequent Akt phosphorylation on Thr308 by PDK1 [45]. Moreover, mTORC2 plays a role in cytoskeleton organization by controlling actin polymerization [46] and phosphorylates protein kinase C (PKC) α [44]. Another down-stream target of mTORC2 is serumand glucocorticoid-induced protein kinase 1 (SGK1) [47]. The oncogenetic role of mTORC2 has been recently highlighted by an investigation that documented the importance of mTORC2 in the development and progression of prostate cancers induced in mice by PTEN loss [48].

Akt and mTORC1/2 are linked to each other via positive and negative regulatory feedback circuits, which restrain their simultaneous hyperactivation through mechanisms which involve p70S6K and PI3K. Assuming that an equilibrium exists between mTORC1 and mTORC2, when mTORC1 is formed, it antagonizes the formation of mTORC2 and reduces Akt activity. Indeed, once mTORC1 is activated through Akt, the former elicits a negative feedback loop for inhibiting Akt activity [34]. This negative regulation of Akt activity by mTORC1 is a consequence of p70S6K-mediated phosphorylation of insulin receptor substrate (IRS) 1 adapter protein, downstream of insulin receptor and/or Insulin-like Growth Factor-1 Receptor (IGF-1R) [49, 50]. Indeed, IRS-1 phosphorylation on Ser307 and Ser636/639 by p70S6K targets the adapter protein to proteasomal degradation [51]. Therefore, at least in principle, inhibition of mTORC1 activity by rapamycin/rapalogs could result in hyperactivation of both Akt and its downstream targets. Such a phenomenon has been documented to occur both *in vitro* and *in vivo* [52, 53]. mTORC1 is capable of downregulating also IRS2 expression by enhancing its proteosomal degradation [54]. Consistently, mTORC1 inhibition by the rapalog, RAD001, increased IRS2 expression and Akt phosphorylation levels in AML cells [55]. Recent work has also highlighted a p70S6K-mediated phosphorylation of Rictor on Thr1135. This phosphorylation event exerted a negative regulatory effect on the mTORC2-dependent phosphorylation of Akt *in vivo* [56]. Thus, both mTORC1 and mTORC2 control Akt activation.

Nevertheless, the extent to which disruption of negative feedbacks mechanism actually limits the therapeutic effects of mTOR inhibitors in cancer patients *in vivo* remains to be determined [57].

### Negative regulation of PI3K/Akt/mTOR signaling

A tight counter-regulation by phosphatases has emerged as a crucial process to control PI3K/Akt/mTOR-dependent signaling. PTEN is a dual specificity lipid/protein phosphatase that preferentially removes the 3′-phosphate mainly from PtdIns (3,4,5)P_3_ but is also active on phosphatidylinositol 3,4 bisphosphate [PtdIns (3,4)P_2_], thereby antagonizing network signaling [58, 59]. PTEN silencing or inactivating mutations have been detected in a wide variety of human neoplasias (including prostatic and endometrium carcinomas, glioblastomas, melanoma, and T-cell acute lymphoblastic leukemia [60]) and this results in Akt/mTOR up-regulation. SHIP-1 and SHIP-2 (for Src homology domain-containing inositol phosphatase) are phosphatases capable of removing the 5-phosphate from PtdIns (3,4,5)P_3_ to yield PtdIns (3,4)P_2_ [61]. An important role for SHIP-1 in normal hematopoiesis has been recently described [62, 63]. PP2A, which is now considered to be an oncosuppressor, down-regulates Akt activity, through dephosphorylation of Thr308 [64]. Thr308 and Ser473 residues of Akt are also targeted by the two isoforms (1 and 2) of PH domain leucine-rich repeat protein phosphatase (PHLPP) [65].

### Activation of PI3K/Akt/mTOR signals in AML

From 50% to 80% of patients with AML display Akt phosphorylated on either Thr308 or Ser473 (or both) [66-71]. Both the disease-free survival and the overall survival were significantly shorter in AML cases where pathway up-regulation was documented [70, 72-74]. Poor prognosis of AML patients with elevated PI3K/Akt/mTOR signaling could be also related to the fact that this pathway controls the expression of the membrane ATP-binding cassette (ABC) transporter, multidrug resistance-associated protein 1, which extrudes chemotherapeutic drugs from leukemic cells and is usually associated with a lower survival rate [75, 76].

Nevertheless, a more recent report has highlighted that constitutive activation of PI3K/Akt/mTOR signaling could be a favourable prognostic factor in de novo cases of AML. One hypothesis for the lower relapse rate in patients with enhanced PI3K/Akt/mTOR signaling is that it could drive immature leukemic cells (LSCs and CFU-L) into S phase, thus rendering them more susceptible to polychemotherapy [77].

**Fig. 3.**
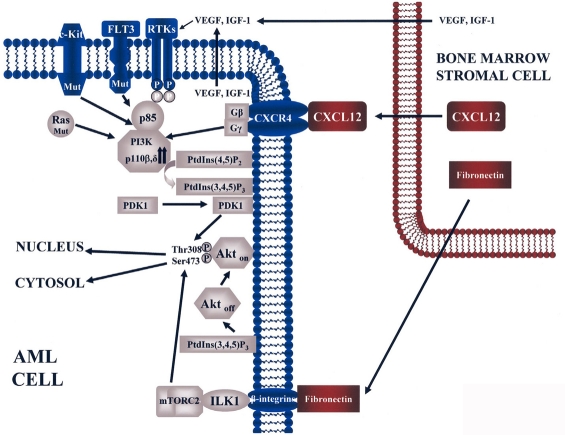
Constitutive activation of PI3K/Akt signaling in AML cells. In this cartoon, mutated (Mut) C-Kit, FLT3, or Ras, and autocrine/paracrine secretion of growth factors (VEGF, IGF-1) impinge upon increased levels of p110β and/or p110δ PI3K. This results in high levels of PtdIns (3,4,5)P_3_ synthesized at the plasma membrane from PtdIns (4,5)P_2_. PtdIns (3,4,5)P_3_ recruits at the plasma membrane both PDK1 and inactive Akt (Akt off). PDK1 phoshorylates Akt on Thr308, whereas phosphorylation on Ser473 is driven by mTORC2. These two phosphorylative events fully activates Akt (Akt on). Bone marrow stromal cells secrete CXCL12 and fibronectin. Fibronectin, by interacting with β integrins, could activate ILK which, in turn, stimulates mTORC2 activity on Ser473 Akt. CXCL12 binds its receptor CXCR4, a GPCR which results in increased PI3K activity. Bone marrow stromal cells could also secrete VEGF and IGF-1. Activated Akt migrates to both nucleus and cytosol to phosphorylate its substrates.

Causes of PI3K/Akt/mTOR signaling up-regulation in AML may be the result of several factors, including activating mutations of Fms-like tyrosine kinase 3 (FLT3) receptor [71] and c-Kit tyrosine kinase receptor [78], N- or K-Ras mutations [79], PI3K p110β and/or δ overexpression [80-82], low levels of PP2A [70], autocrine/paracrine secretion of growth factors such as IGF-1 [82-84] and VEGF [85, 86]. Overexpression of PDK1 has been reported in 45% of a cohort of 66 AML patients, however it was related to PKC hyperphosphorylation, while the relationship (if any) with Thr308 Akt up-regulation was not investigated [87]. Interactions between leukemic cells and bone marrow stromal cells through CXCR4 (a GPCR which is abundantly expressed on leukemic cell surface where it is up-regulated by hypoxic conditions [88, 89]) and its physiological ligand, CXCL12, produced by stromal cells [89, 90], could result in PI3K/Akt/mTOR activation [91]. Furthermore, interactions between β1 integrins on AML cells and stromal fibronectin could lead to pathway activation [92, 93], possibly through up-regulation of integrin-linked kinase 1 (ILK1) which is involved in Akt phosphorylation on Ser473 in a PI3K-dependent manner in AML cells [94]. The ability of ILK1 to function as a Ser473 Akt kinase could be related to the fact that ILK1 interacted with Rictor and was required for Akt phosphorylation by mTORC2 on Ser473 [95]. Possible causes of pathway activation in AML cells are highlighted in **Figure 3**.

No activating mutations in p110α PI3K [96] or Akt1 PH domain [70, 97] have been detected so far in AML patients. Although PTEN is deleted in many solid cancers and T-cell acute lymphoblastic leukemia, PTEN deletion is extremely rare in AML [66, 69, 70]. PTEN can be inactivated by post-translational mechanisms, including phosphorylation at the COOH-terminal regulatory domain. This phosphorylative event stabilizes PTEN molecule but makes it less active towards PtdIns (3,4,5)P_3_, thus resulting in Akt up-regulation [98]. PTEN phosphorylation has been reported in AML patients where it was significantly associated with high levels of p-Akt and with shorter overall survival [99]. However, subsequent studies could not confirm these findings [70, 74]. A reassessment of the PTEN role in AML could be important, as in mice, hematopoietic stem cells without functional PTEN, began multiplying rapidly, showed diminished self-renewal capacity, and started to move out of the bone marrow, colonizing distant organs, and originating a leukemic-like disease [100, 101]. Of note, these effects were mostly mediated by mTOR, as rapamycin not only depleted LSCs, but also restored normal hematopoietic stem cell function [101].

It is conceivable that several concomitant extrinsic and intrinsic causes converge to activate PI3K/Akt/mTOR signaling in AML patients, even if this fundamental issue has not been thoroughly investigated. Indeed, in the only published study, it was demonstrated that, in a small cohort of patients, overexpression of PI3K p110δ [81] could coexist with activating FLT3 and Ras mutations. It has also been reported that mTORC1 activation was independent of PI3K/Akt activity in AML patients [55]. In some AML cases, it has been documented that either MEK/ERK 1/2 [102] or Lyn signaling [103] could be up-stream of mTORC1. TSC2 gene expression was found to be down-regulated in AML patients, most likely due to promoter hypermethylation. However, it is not known if it impinged on mTORC1 activation [104].

It should be emphasized here that PI3K/Akt/mTOR network up-regulation has been detected not only in the bulk of the AML blasts, but also in LSCs transplanted in non-obese diabetic/severe combined immunodeficiency (NOD/SCID) mice, where it exerted a powerful pro-survival effect. This finding suggests that therapeutic targeting of this pathway has the potential for eradicating AML [105].

### Targeting PI3K/Akt/mTOR module in AML

Either used alone or in combination with other drugs, PI3K/Akt/mTOR signaling inhibitors have been proven useful for down-regulating cell proliferation and inducing apoptosis in pre-clinical settings of AML, using cell lines or animal models. However, clinical trials of these compounds are limited. We shall now highlight some compounds which have been used for targeting PI3K/Akt/mTOR signaling in AML cells.

#### PI3K inhibitors

Wortmannin and LY294002 are the best characterized PI3K inhibitors that have been widely used as research tools to elucidate the role of PI3K/Akt/mTOR signaling in various tumor cells. Both inhibitors are cell-permeable and low molecular weight compounds. Wortmannin is a natural metabolite produced by *Penicillium wortmanni* and inhibits all class PI3K members with a 50% inhibitory concentration (IC_50_) *in vitro* of 2-5 nM, while inhibiting other with histone deacetylase inhibitors [117] or pro-apoptotic kinases [mTOR, DNA-dependent protein kinase (DNA-PK), and ataxia telangiectasia mutated kinase] with higher IC50 values [106]. It is interesting that DNA-PK was found to phosphorylate Akt on Ser473 under conditions of DNA damage [107].

LY294002 is a flavonoid-based synthetic compound and inhibits PI3K with an IC_50_ of 1-20 μM. However, LY294002 perifosine and UCN-01 (a staurosporine derivative which blocks not only PI3K activity but also mTOR, DNA-PK, Pim kinase, polo-like kinase, and CK2 to the same extent as PI3K [106]. Both wortmannin and LY294002 bind to the p110 catalytic subunit of PI3K, leading to the blockade of ATP bound to the active portion. PI3K inhibition with LY294002 is reversible and ATP-competitive while wortmannin irreversibly inhibits PI3K in a non-ATP-competitive manner [106].

Wortmannin and LY294002 have been used in preclinical models of AML where they displayed powerful cytotoxic effects *in vitro* [66, 79, 108, 109]. Since the insolubility in aqueous solutions and high toxicity of both inhibitors precluded their clinical application, efforts to develop PI3K inhibitors more suitable for clinical use are currently underway [110].

Several selective inhibitors of p110 PI3K isoforms are now available [111]. IC87114 is a compound that selectively inhibits the p110δ isoform of PI3K. IC87114 down-regulated p-Akt and p-FOXO3a, reduced proliferation, and induced apoptosis in AML primary cells overexpressing p110δ?PI3K. Moreover, it synergized with etoposide [81]. In primary APL cells, both IC87114 and TGX-115 (a p110β PI3K-selective inhibitor) triggered apoptosis in the presence or in the absence of the differentiating agent, ATRA [29].

Conceivably, the use of selective PI3K isoform inhibitors could be associated with less undesirable side effects than the use of broad spectrum PI3K inhibitors [111]. For example, it is established that insulin control of glucose homeostasis is mainly mediated through p110α PI3K [112] and, to a much lower extent, by p110β PI3K [113].

#### Akt inhibitors

Perifosine is a zwitterionic, water soluble, synthetic alkylphosphocholine with oral bioavailability that inhibits Akt phosphorylation through interaction with the Akt PH domain, resulting in disruption of its membrane targeting. Interestingly, recent evidence has documented that perifosine targets both mTORC1 and mTORC2 activity by down-regulating the levels of mTOR, raptor, rictor, p70S6K, and 4E-BP1, owing to their enhanced degradation [114]. Perifosine reduced cell proliferation and induced apoptosis accompanied by Akt dephosphorylation in a wide variety of neoplasias, including AML [115]. Perifosine synergized with etoposide in AML blasts, and reduced the clonogenic activity of CD34^+^ cells from leukemic patients, but not from healthy donors [116]. Moreover, perifosine synergized with histone deacetylase inhibitors [117] or pro-apoptotic TRAIL (TNF-related Apoptosis Inducing Ligand) in AML cell lines and primary cells displaying Akt constitutive activation [118]. However, perifosine also targeted the MER/ERK 1/2 pro-survival pathway and activated pro-apoptotic JNK, [116-120] therefore it could not be considered specific for the Akt pathway. A phase 1 clinical trial combining perifosine and UCN-01 (a staurosporine derivative which inhibits PDK1) (NCT00301938) and a phase II clinical trial with perifosine alone (NCT00391560) have been performed in patients with refractory/relapsed AML, but the results have not yet been disclosed.

Akt-I-1/2, a synthetic reversible allosteric inhibitor, is an Akt1/Akt2 isoform-specific inhibitor that forms a PH domain-dependent inactive conformation with Akt1 and Akt2 [121]. Akt-I-1/2 inhibited cell proliferation and clonogenic properties, and induced apoptosis in AML cells with high-risk cytogenetic changes/abnormalities [70]. However, it is at present unknown which Akt isoforms are expressed by AML blasts.

#### mTOR inhibitors

mTOR inhibitors are by far the most developed class of compounds which target the PI3K/Akt/mTOR pathway. They include: rapamycin (sirolimus, a macrolide derived from the bacterium *Streptomyces hygroscopicus*, originally discovered in a soil sample collected on Easter Island) and its derivatives CCI-779 (temsirolimus), RAD001 (everolimus), and AP23573 (deforolimus) [122]. Temsirolimus was approved by US Food and Drug Administration in 2007 for the first-line treatment of poor prognosis patients with advanced renal cell carcinoma. The overall survival of treated patients was increased by nearly 50% (~3 months) relative to the control group [123]. Some clinical benefits of rapamycin/rapalogs have been reported also against endometrial carcinoma and mantle cell lymphoma, however, the overall objective response rates in major solid tumors have been modest [124].

Rapamycin and rapalogs do not target the catalytic site of mTORC1, but rather bind its immunophilin, FK506 binding protein 12 (FKBP12) (**Figure 2**). The rapamycin/FKBP12 complex then binds mTORC1 and inhibits downstream signaling events [125]. Thus, rapamycin and rapalogs act as allosteric mTORC1 inhibitors. Recent evidence has documented that complex formation with FKBP12 is not an absolute requirement for repression of mTORC1 activity by rapamycin/rapalogs, however, in the absence of FKBP12, the drugs display a 100 to 1000-fold lower potency than in the presence of the immunophilin [126]. Available data suggest that rapamycin treatment, over long time periods, also targets mTORC2 [127]. Accordingly, both CCI-779 and RAD001 (10-20 nM) inhibited Akt phosphorylation on Ser473 in AML cells *in vitro* and in patients *in vivo* after a 24 h incubation, through suppression of the mTORC2 assembly [128]. In contrast, it has been documented that RAD001 (10 nM for 24 h) increased Akt phosphorylation *in vitro* on Ser473 in AML samples displaying constitutive PI3K/Akt activation [55]. Since a neutralizing monoclonal antibody to the IGF-1R α-subunit, reversed the RAD001-induced increase of Akt phosphorylation and RAD001 treatment led to a significant increase in IRS2 protein expression, it was concluded that p-Akt upregulation could be explained by the existence of an IGF-1/IGF-1R autocrine loop, as well as by increased expression of IRS2. At present, it is not easy to reconcile these contradictory findings.

Rapamycin had only a modest effect on primary AML cell survival in liquid culture, however, it markedly down-regulated AML blast clonogenicity while sparing normal hematopoietic precursors [129]. Accordingly, others have reported that rapamycin led to only a slight decrease in AML blast survival in short term cultures, whereas in long term cultures the effect was more pronounced [105]. These results suggested that the target of rapamycin is the proliferating contingent of the leukemic clone, rather than the bulk of AML blasts which are predominantly blocked in the G0/G1 phase of the cell cycle.

However, rapamycin cytotoxicity in short term cultures could be dramatically increased by co-treatment with etoposide. Importantly, etoposide toxicity on CD34^+^ cells from healthy donors was not enhanced by addition of rapamycin. Of note, co-incubation with rapamycin enhanced etoposide-mediated decrease in the engraftment of AML cells in NOD/SCID mice, suggesting the drugs also targeted putative LCSs [105].

The rapalog RAD001 synergized with both ATRA and histone acetylase inhibitors in inducing growth arrest and differentiation of APL cell lines [130, 131].

A few phase I/II clinical trials with rapamycin and rapalogs have been performed in patients with relapsed/refractory AML. Rapamycin induced a partial response in 4 of 9 adult patients with de novo or secondary AML, who displayed activation of mTORC1 signaling, as documented by increased levels of p-p70S6K and p-4E-BP1 [129]. RAD001 has been evaluated in a phase I clinical trial in patients with relapsed/refractory hematologic malignancies, including AML [132]. However, no AML patients achieved a complete or even partial response. AP23573 has been tested in a phase II study in 22 patients with AML [133]. Only one patient displayed an objective hematological improvement, consisting of normalization of neutrophils. A significant reduction in mTORC1 activity was observed in response to the drug, as documented by decreased p-4E-BP1 levels. A recent phase I study in which rapamycin was combined with MEC (mitoxantrone, etoposide, cytarabine) polychemotherapy failed to demonstrate any synergistic effect of the combination in relapsed/refractory AML patients, even if proof of rapamycin biological activity *in vivo* was detected, consisting in the dephosphorylation of p70S6K [134]. Several clinical trials with rapamycin/rapalogs combined with chemotherapeutic agents are now underway in AML patients [135].

Moreover, a phase I study has recently documented the efficacy, in elderly AML patients, of the combination etoposide and tipifarnib (R11577, an FTI). Intriguingly, the effect of tipifarnib was not always related to Ras inhibition, but rather to inhibition of Rheb farnesylation and, consequently, of mTORC1 signaling, as documented by decreased levels of p-p70S6K and of its substrate, p-S6 [136].

#### Dual PI3K/mTOR inhibitors

The rationale for using dual PI3K/mTOR inhibitors is that mTORC1 allosteric inhibitors, such as rapamycin/rapalogues, could hyperactivate Akt through p70S6K/PI3K, as discussed earlier in this review. Moreover, it is now emerging that rapamycin/rapalogs have only modest efficacy on total translation rates, and the effects are cell-type specific. In contrast, small molecules designed for inhibiting the catalytic site of mTOR, were much more effective in this respect, especially in cancer cells [137-141]. Such a phenomenon has been recently reported to occur also in AML cells, where rapamycin was unable to block protein synthesis, owing to a failure in inducing 4E-BP1 dephosphorylation [142]. Furthermore, in some AML cases, mTORC1 activity does not seem to be under the control of PI3K/Akt, despite concomitant PI3K/Akt activation [103]. Therefore, the use of a single inhibitor which targets both PI3K and mTORC1 catalytic sites could present substantial advantages over drugs which only target either PI3K/Akt or mTORC1. PI-103 is a pyridonylfuranopyrimidine class synthetic molecule that represses the activity of both class IA and IB PI3Ks, as well as of mTORC1/mTORC2 [143, 144]. Two papers have documented the efficacy of PI-103 in pre-clinical settings of AML. It has been reported that PI-103, which itself displayed only modest pro-apoptotic activity, acted synergistically with Nutlin-3 (an MDM2 inhibitor) [145, 146], to induce apoptosis in a wild-type p53-dependent fashion in AML cell lines and primary cells [147]. Another group demonstrated that PI-103 was mainly cytostatic for AML cell lines. However, in AML blast cells, PI-103 inhibited leukemic proliferation and CFU-L clonogenicity, induced mitochondrial apoptosis, and synergized with etoposide [148]. Of note, PI-103 was not apoptogenic in CD34+ cells from healthy donors and had only moderate effects on their clonogenic and proliferative activities. Since either RAD001 or IC87114 did not induce apoptosis in AML primary cells, it was concluded that dual-targeted therapy against PI3K/Akt and mTOR with PI-103 may be of therapeutic value in AML [148].

Nevertheless, it is conceivable that the new frontier in mTOR inhibition will be represented by the second generation, ATP-competitive mTOR inhibitors which bind the active site of both mTORC1 and mTORC2 [137-140]. These drugs target mTOR signaling functions in a global way, so that they are expected to yield a deeper and broader antitumor response in the clinic. However, global inhibition of mTOR is expected to be accompanied by greater toxicity to normal cells [149].

## CONCLUSIONS

In this review, we have documented that the PI3K/Akt/mTOR pathway influences proliferation, survival, and drug resistance of AML cells. However, there still are many unresolved problems regarding the relevance of PI3K/Akt/mTOR pathway up-regulation and its druggability in AML patients. We have a very limited knowledge of the downstream targets (genes/proteins) of this pathway in AML cells. Therefore, more detailed investigations of these targets are highly desirable. Indeed, data emerging from gene expression and proteome/phosphoproteome analysis could pave the way for functional studies which could then provide valuable information for improving future therapeutic strategies. At present, we do not know what is the most effective target in the pathway, and whether combinations of horizontal or vertical blockade of the signaling cascade may be more effective than blocking at a single node [150].

As with all molecularly targeted approaches, pharmacodynamic markers are necessary to direct therapeutic development of PI3K/Akt/mTOR inhibitors. Hence, clinical trials should examine the inhibitor effects on PI3K/Akt/mTOR targets to establish the best predictor of response [151]. However, no predictive markers for AML patients with a high probability of responding to PI3K/Akt/mTOR inhibition, or biomarkers of dose/efficacy, have been validated. Quantitative flow cytometry appears particularly well suited for this kind of analysis, because it offers obvious advantages over other techniques (western blot, for example), including quickness, a much lower number of cells required to perform the assay, and the possibility of identifying different subclones in the leukemic population by co-immunostaining with multiple antibodies to surface antigens. Accordingly, flow cytometry is rapidly becoming the choice analytical technique to study PI3K/Akt/mTOR pathway activation in AML patients [70, 133, 152, 153]. Another promising quantitative technique requiring a limited number of cells, which has been already applied to the study of AML patients samples, is represented by reverse-phase protein arrays [74].

It is highly unlikely that inhibition of a single signaling pathway will achieve long-lasting remissions or cure in AML, especially for refractory/relapsed patients. However, combining PI3K/Akt/mTOR inhibitors with conventional chemotherapy drugs, differentiation inducers (ATRA and/or arsenic trioxide), or innovative (e.g. TRAIL) agents could be a very effective therapeutic option for AML patients, as indicated by results obtained in pre-clinical settings.

The spectacular effect of Bcr-Abl tyrosine kinase inhibitors, such as imatinib for the treatment of chronic myelogenous leukemia (CML) patients in the chronic phase of the disease [154], has fed optimism that modulators of signal transduction networks might be very effective also in other types of cancer. However, clinical trials performed with small molecules targeting the PI3K/Akt/mTOR pathway have mostly given a disappointing outcome. This fact has led to the suggestion that imatinib success in CML may be the exception and not the rule, because imatinib is one of the few examples of a drug targeting the anomaly which constitutes the underlying pathologic event in the formation of the disorder [155]. Human cancers are known to evolve through a multistage process which can extend over a period of several years. Therefore, they progressively accumulate mutations and epigenetic anomalies in expression of multiple genes [156]. As a consequence, neoplastic disorders are characterized by multiple signaling abnormalities and the deregulated pathways are extremely redundant. Furthermore, the hierarchy of anomalies has not been established in many tumors. Therefore, it could be very difficult to find the right target or combinations of target.

AML is no exception to this rule. However, the continuous development of molecularly targeted drugs displaying higher selectivity, coupled with additional mechanistic studies and advances in profiling the signaling networks of cancer cells, should make it possible to exploit deregulation of the PI3K/Akt/mTOR cascade to achieve more effective and less toxic therapies for AML.
